# Single amino acid substitution in RdRp reduces viral recombination frequency of NADC30-like porcine reproductive and respiratory syndrome virus type 2

**DOI:** 10.1371/journal.ppat.1014500

**Published:** 2026-08-05

**Authors:** Xingyang Cui, Hao Song, Xinyi Huang, Dasong Xia, Yongbo Yang, Xiaoxiao Tian, Tao Wang, Guoqing Liu, Zameel Saleem, Haiwei Wang, Tongqing An

**Affiliations:** 1 State Key Laboratory of Animal Disease Control and Prevention, Harbin Veterinary Research Institute, Chinese Academy of Agricultural Sciences, Harbin, China; 2 College of Animal Science, Wenzhou Vocational College of Science and Technology, Wenzhou, China; 3 Heilongjiang Provincial Key Laboratory of Veterinary Immunology, Harbin, China; Kobenhavns Universitet, DENMARK

## Abstract

Porcine reproductive and respiratory syndrome (PRRS) is one of the most significant diseases, causing tremendous economic losses to the global swine industry. Accumulating evidence indicates that recombination between PRRSV-2 strains, particularly those involving NADC30-like PRRSV-2 variants, has been responsible for outbreaks across several countries. However, the key proteins or amino acids associated with PRRSV-2 recombination remain unclear. In this study, two representative PRRSV-2 strains (the NADC30-like HeB108 strain from lineage 1 and the HP-PRRSV HuN4 strain from lineage 8) were serially passaged in the presence of ribavirin. Amino acid substitutions associated with ribavirin resistance were identified in the RNA-dependent RNA polymerase (RdRp) and helicase of the passaged viruses. Using the infectious clones PRRSV-2 HeB108 and HuN4, ribavirin-resistant mutants with single or multiple amino acid substitutions were generated. Notably, the mutants HeB108-VGSS and HuN4-TNII, with multiple amino acid substitutions, showed higher fidelity and lower pathogenicity in infected piglets than their parental viruses. Furthermore, the potential decreased recombination risk correlated with increased polymerase fidelity. In an inter-lineage co-infection assay on primary alveolar macrophages, the I360V substitution in RdRp reduced the recombination frequency of NADC30-like PRRSV-2 HeB108 as determined by both Sanger sequencing and nanopore long-read RNA sequencing. The results identified a key amino acid associated with viral recombination in NADC30-like PRRSV-2 and provide an editing target for developing safer vaccines that are less prone to recombination.

## Author summary

PRRSV-2 has undergone frequent recombination, making it difficult to prevent and control. However, the relevant mechanisms remain unknown due to the complexity of PRRSV-2 recombination. This study used the nucleoside analogue ribavirin to screen for PRRSV-2 resistant strains, identify key amino acid substitution sites, and demonstrate that ribavirin-resistant substitutions can reduce viral recombination rates, indicating the essential roles of RdRp and/or helicase in PRRSV-2 recombination. The potential recombination risk was inversely correlated with RdRp fidelity. More interestingly, the I360V substitution in RdRp was confirmed to be associated with viral recombination frequency of NADC30-like PRRSV-2. This is a more detailed study of the possible recombination mechanisms of PRRSV-2. The results provide insights into viral recombination, and the key residue involved in polymerase fidelity is a potential target for future vaccine development.

## Introduction

Porcine reproductive and respiratory syndrome (PRRS) is a highly contagious and fatal infectious disease characterized by reproductive failure in sows and respiratory symptoms in pigs, causing enormous economic losses in pork production worldwide [1,2]. PRRS was first reported in the United States in 1987, and is now detectable in most pig-raising countries [3]. PRRS virus (PRRSV), an enveloped single-stranded positive-sense RNA virus, belongs to the genus *Betaarterivirus*, family *Arteriviridae*, and order *Nidovirales* [4]. PRRSV is divided into two species: *Betaarterivirus suid* 1 (formerly known as PRRSV-1), represented by the Lelystad strain [5], and *Betaarterivirus suid* 2 (formerly known as PRRSV-2), represented by the VR-2332 strain [6]. The PRRSV-2 genome is approximately 15 kb in length with a 5’-untranslated region and 3’-untranslated region with a poly (A) tail, which contains at least 10 open reading frames (ORFs), including ORF1a, ORF1b, ORF2a, ORF2b, ORF3–7, and ORF5a [7]. ORF1a and ORF1b encode polyproteins pp1a and pp1ab, which are cleaved into at least 14 non-structural proteins (nsps). Proteolytic cleavage of pp1ab generates nsp9 to nsp12, which participate in the transcription and replication of the viral genome. The viral replication–transcription complex (RTC) is composed of multiple nonstructural proteins, among which nsp9 and nsp10 serve as key components. The RdRp nsp9 plays a critical role in viral RNA transcription and replication [8]. The nsp10 protein is a helicase and can drive viral genome replication, viral particle generation, and subgenomic RNA synthesis [9].

PRRSV-2 has high genetic diversity owing to nucleotide mutations and recombination. Recombination occurs during viral replication, sometimes resulting in chimeric viruses that are more infectious than their parent viruses [10,11]. Many PRRSV-2 variants with altered pathogenicity have been produced via recombination [12–15]. Furthermore, recombinant PRRSV-1 and PRRSV-2 can evade the adaptive immune response induced by existing PRRS vaccines [12,14,15], thereby complicating the PRRS epidemic. Notably, frequent recombination poses the risk of virulence reversion in PRRSV-1 and PRRSV-2 modified live vaccines (MLV) [15–18].

Based on ORF5 gene diversity, PRRSV-2 strains are divided into 11 lineages (L1-L11) [19,20]. A highly pathogenic PRRSV-2 (HP-PRRSV) belonging to the L8 of PRRSV-2 has emerged in China, Thailand and Vietnam since 2006, and the HP-PRRSV has become dominant in China and some Asian countries [21,22]. In 2013, NADC30-like PRRSV-2, belonging to the L1 lineage, was introduced into China from North America and spread rapidly across the country [23]. The co-circulation of multiple PRRSV-2 lineages leads to frequent generation of recombinant variants. The NADC30-like PRRSV-2 strain is recognized as the major parent among PRRSV-2 recombinants [14,24]. However, the underlying mechanisms driving its dominance in recombination events remain unknown.

Viral recombination and error-prone RNA-dependent RNA polymerase (RdRp) are the two predominant factors contributing to the diversity of RNA viruses. RNA viruses have the highest mutation rates, ranging from 10^-4^ to 10^-6^ mutations per round of genome replication [25]. The lack of RdRp proofreading leads to high mutation rates, which control viral evolution and immune escape [26,27]. Nucleoside analogue-resistant amino acid substitutions in RdRp could affect polymerase fidelity. In most cases, viral mutants with reduced nucleoside analogue sensitivity exhibit increased polymerase fidelity [28–30]. Co-infection of a single cell could generate recombinant viruses from two closely related viruses [10]. Recent studies have shown that changes in fidelity affect recombination efficiency [31–33]. Therefore, increasing RdRp fidelity can reduce recombination efficiency, and vice versa. The relationship between RdRp fidelity and viral recombination has been confirmed in Senecavirus, Enterovirus, and Sindbis virus [31–33]. Besides speed and fidelity, the inherent biochemical properties of RdRp also determine the mechanism and efficiency of the recombination [34]. In addition, studies have shown that viral helicases participate in viral recombination, for example, the helicase domain of the potato virus X replicase is involved in homologous RNA recombination [35], and mutations in the helicase can alter the sites of RNA-RNA recombination in the brome mosaic virus [36].

At present, PRRSV-2 remains a significant threat to the pig industry due to its high recombination rate. However, the recombination-associated factors have not yet been identified. In the present study, two representative strains, L1 NADC30-like PRRSV-2 and L8 HP-PRRSV, were used for ribavirin pressure screening. The ribavirin-resistance-related substitutions in viral RdRp and helicase, two key replication-related enzymes [9,37,38], were identified and their contributions to viral replication fidelity and recombination frequency were evaluated *in vivo* or *in vitro*. This study identified a specific amino acid substitution in the RdRp of PRRSV-2 associated with viral recombination and modulating viral replication fidelity. This finding provides a foundation for generating a novel PRRSV-2 vaccine resistant to recombination.

## Results

### Amino acid substitutions in nsp9 and nsp10 of ribavirin-resistant PRRSV-2

Ribavirin is a nucleoside analogue and a viral RNA polymerase inhibitor with broad-spectrum antiviral activity. Ribavirin cytotoxicity in Marc-145 cells was evaluated. Cell viability remained unaffected even at concentrations up to 400 μM. Subsequently, viruses were passaged in Marc-145 cells in the presence of 100 or 200 μM ribavirin. Under these conditions, PRRSV-2 titers did not decrease significantly. Whereas, at concentrations of 250, 300, 350 and 400 μM, the viral titer began to decrease significantly (S1 Fig). Therefore, 250 μM ribavirin was selected as the optimal initial screening concentration for serial passaging of PRRSV-2. L1 NADC30-like HeB108 and L8 HP-PRRSV HuN4, were selected for ribavirin pressure screening. These strains were preserved in our laboratory. PRRSV-2 HeB108 and HuN4 strains harvested at passages P5, P10, P15, P20, P25, and P30 were sequenced to identify nucleotide changes resulting in amino acid substitutions in nsp9 and nsp10. Sequence comparisons of PRRSV-2 screened with or without ribavirin treatment revealed that most mutations were synonymous, and only a few nucleotide changes resulted in amino acid substitutions. Results showed that I360V of nsp9 and S62G, G327S, and N406S of nsp10 occurred in HeB108 (S1 Table). Several substitutions, including A286T, D491N, and T544I in nsp9, and T205I in nsp10, were observed in HuN4 (S2 Table). These results suggested that these amino acid substitutions were associated with ribavirin resistance.

### Location of ribavirin-resistance-related mutations in nsp9 and nsp10

**Fig 1:**
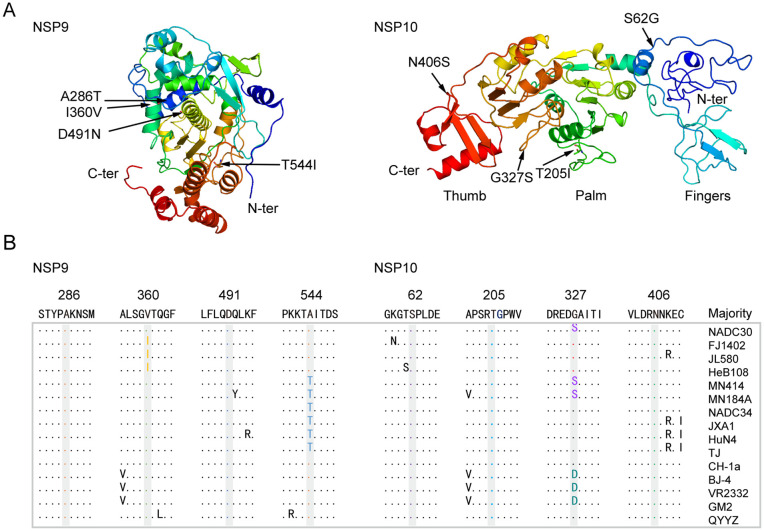
Location of ribavirin-resistant mutation in the RdRp and helicase of PRRSV-2. **(A)** The ribavirin-resistant mutations in the nsp9 (RdRp) and nsp10 (helicase). The residues at positions 286, 360, 491, and 544 in nsp9. The residues at positions 62, 205, 327, and 406 in nsp10. **(B)** Alignment of nsp9 and nsp10 amino acid sequences revealed the conservation of different mutation sites. Colors indicate the locations of mutations screened with ribavirin.

The residues A286, D491, T544, and I360 were located near the RNA-binding pocket in a 3D model of the nsp9 protein. Notably, in nsp10, T205 and G327 were in the palm domain, N406 was in the thumb domain, and S62 was in the finger domain (Fig 1A), suggesting that these substitutions potentially affect viral replication. Sequence alignment revealed that A286 and D491 in nsp9, and S62, T205, and N406 in nsp10 were highly conserved across different PRRSV-2 strains (Fig 1B).

### Rescue of ribavirin-resistant PRRSV-2 mutants based on infectious clones

**Fig 2:**
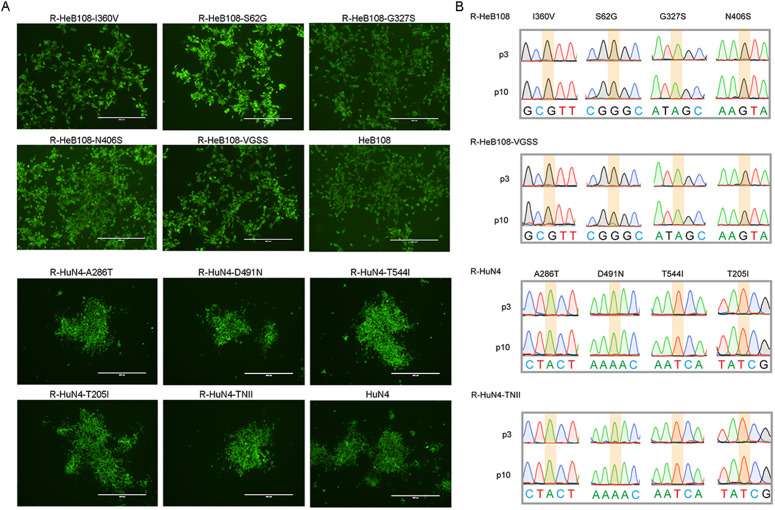
Identification of the ribavirin-resistant PRRSV-2 mutants. **(A)** Identification by immunofluorescence assay. Recombinant and parental plasmids (4 μg) were transfected into Marc-145 cells. At 5 d post-transfection, the transfected cells were fixed and stained with the monoclonal antibody 3F7 against PRRSV-2, followed by incubation with Alexa Fluor 488-conjugated goat anti-mouse IgG. Scale bar, 400 μm. **(B)** Genetic stability of the mutation sites in the P3 and P10 generations.

According to the above mutation sites selected in the presence of ribavirin, eight individual mutants (R-HuN4-A286T, R-HuN4-D491N, R-HuN4-T544I, R-HuN4-T205I, R-HeB108-I360V, R-HeB108-S62G, R-HeB108-G327S and R-HeB108-N406S) and two combined mutants (R-HuN4-TNII and R-HeB108-VGSS) were constructed and rescued based on the HeB108 and HuN4 infectious clones, respectively. Rescued mutant viruses were confirmed by cytopathic effect (CPE), immunofluorescence assay (IFA), and Sanger sequencing (Fig 2A). In addition, the mutations were stable during 10 serial passages in Marc-145 cells (Fig 2B).

### Identification of PRRSV-2 variants with reduced sensitivity to ribavirin

**Fig 3:**
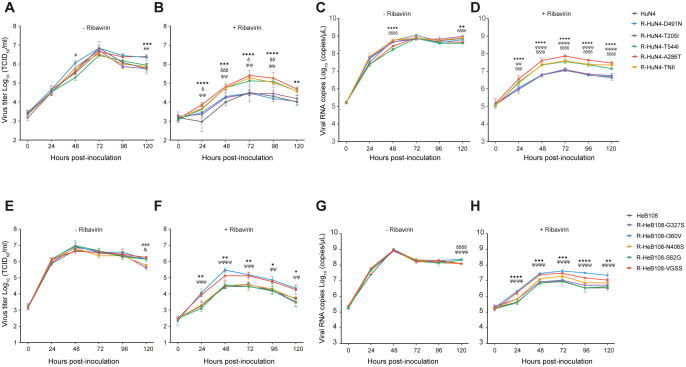
Growth kinetics of the ribavirin-resistant PRRSV-2 mutants. Growth properties of HuN4‑derived **(A–D)** and HeB108‑derived **(E–H)** ribavirin‑resistant mutants in Marc‑145 cells in the absence (-Ribavirin) or presence (+Ribavirin) of 300 μM ribavirin. Confluent Marc‑145 cell monolayers in 24-well plates were infected with the indicated ribavirin‑resistant mutants at an MOI of 1. Each data point represents the mean value of three replicates with SD. The symbols in panels A–H denote the statistical significance of differences between groups: *, *P* < 0.05, **, *P* < 0.01, ***, *P* < 0.001, ****, *P* < 0.0001. Specifically, in panels A–D: *, HuN4 vs. R-HuN4-A286T, ^#^, HuN4 vs. R-HuN4-D491N, ^&^, HuN4 vs. R-HuN4-T205I, ^δ^, HuN4 vs. R-HuN4-T544I, ^Ψ^, HuN4 vs. R-HuN4-TNII. In panels E–H: *, HeB108 vs. VGSS, ^#^, HeB108 vs. R-HeB108-G327S, ^&^, HeB108 vs. R-HeB108-N406S, ^δ^, HeB108 vs. S62G, ^Ψ^, HeB108 vs. I360V.

To evaluate whether the identified amino acid substitutions could reduce viral sensitivity to ribavirin, one-step growth curve analyses were performed in the absence or presence of 300 μM ribavirin. Compared with untreated conditions, ribavirin treatment markedly inhibited the replication of both parental and mutant viruses, indicating that ribavirin effectively restricts PRRSV-2 replication (Fig 3A-H). Under ribavirin-free conditions, HuN4-derived mutant viruses exhibited overall similar replication kinetics to the parental virus HuN4. Only at 120 hpi, the viral titers of R-HuN4-D491N and R-HuN4-A286T mutants were slightly higher than those of the parental virus HuN4 (Fig 3A and 3C). For HeB108-derived mutants, only R-HeB108-G327S and R-HeB108-N406S mutants showed slightly higher viral titers than the parental virus HeB108 at 120 hpi, while no significant differences were observed at other time points (Fig 3E and 3G). However, under 300 μM ribavirin treatment, several mutant viruses displayed enhanced replication advantages. Specifically, R-HuN4-A286T, R-HuN4-T544I, and R-HuN4-TNII mutants exhibited significantly higher replication levels than the parental virus HuN4 at all tested time points except 120 hpi (Fig 3B and 3D). Similarly, R-HeB108-I360V and R-HeB108-VGSS mutants showed significantly higher viral titers and RNA copy numbers than the parental virus HeB108 at multiple time points post infection (Fig 3F and 3H). These results demonstrate that amino acid substitutions such as A286T, T544I, and I360V enhance viral replication under ribavirin pressure and reduce PRRSV-2 susceptibility to ribavirin.

### Combined mutants showed lower pathogenicity to piglets

**Fig 4:**
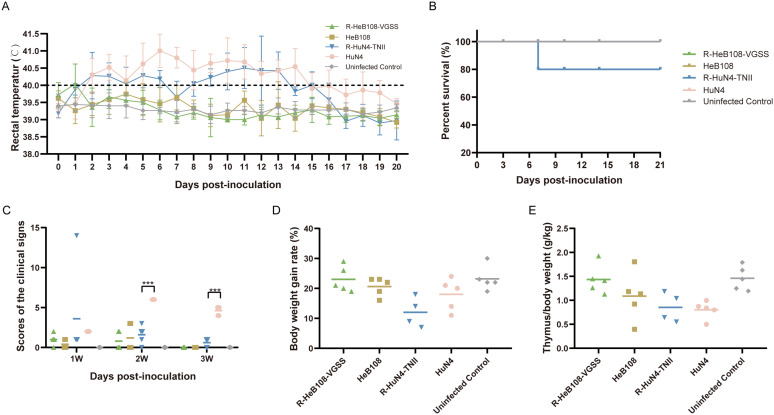
Clinical symptoms of piglets infected with R-HeB108-VGSS, R-HuN4-TNII, and their parental viruses. **(A)** Rectal temperatures of piglets. The cut-off value of fever was set at 40.0 ℃. The percent survival **(B)**, clinical sign scores of piglets **(C)**, average daily weight gain rate **(D)**, and thymus/body weight (g/kg) ratio **(E)**, were compared between the infected and uninfected groups. *, *P* < 0.05, ***, *P* < 0.001, significant difference between the infected and uninfected groups. The data are presented as mean ± *SD*.

To explore the pathogenicity of the PRRSV-2 ribavirin-resistant mutants, piglets were infected with R-HeB108-VGSS, R-HuN4-TNII, HeB108 or HuN4. In R-HuN4-TNII- or HuN4- infected piglets, rectal temperature increased gradually from 3 dpi and was maintained for approximately two weeks. HuN4-infected piglets reached a highest temperature of 41.5 ℃ at 6 dpi, whereas piglets infected with R-HuN4-TNII reached a highest temperature of 41 ℃ at 11 dpi. The temperature of HuN4-infected piglets was always higher than that of R-HuN4-TNII-infected piglets (Fig 4A). In contrast, the temperatures of HeB108- and HeB108-VGSS-infected piglets were low, and there were no obvious symptoms of fever. Except for one piglet infected with R-HuN4-TNII, all piglets survived throughout the experimental period (Fig 4B). Regarding the clinical symptom scores, the onset was earlier in both the HuN4 and HeB108 groups than in the R-HuN4-TNII and R-HeB108-VGSS groups (Fig 4C). The average daily weight gain of HeB108-infected piglets was lower than that of HeB108-VGSS-infected piglets (Fig 4D). Moreover, the weight of the thymus of HeB108-infected piglets was lower than that of R-HeB108-VGSS-infected piglets (Fig 4E). The results indicated that the clinical symptoms of ribavirin-resistant mutant-infected piglets were milder than those caused by their parent viruses.

**Fig 5:**
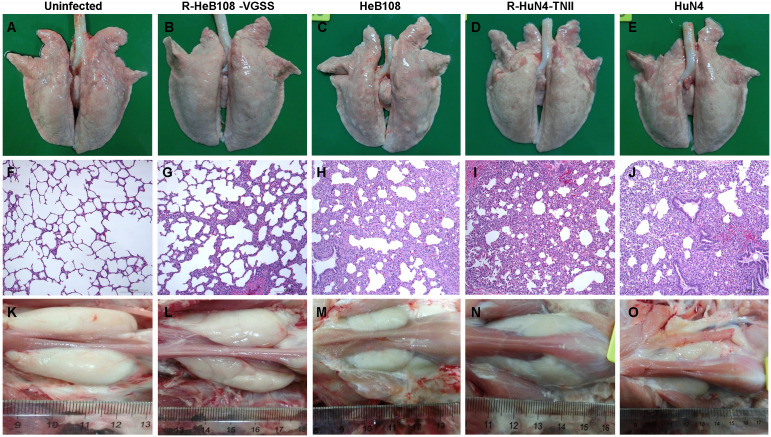
Pathological lesion of the lung and thymus. **(A–E)** Lungs of infected and uninfected piglets, consolidation was observed in the lungs of infected piglets. **(F–J)** Histopathological examination of the lungs of infected piglets. Massive lymphomononuclear cell infiltration was observed in HuN4 infected piglets. **(K–O)** Thymus atrophy caused by R-HuN4 and R-HeB108 was less severe than that caused by parental viruses HuN4 and HeB108.

Histopathological examination revealed more severe lung lesions in piglets infected with HuN4 or R-HuN4-TNII than in those infected with HeB108 or R-HeB108-VGSS (Fig 5A–E). However, lesions caused by the mutant viruses were consistently milder than those induced by their respective parental strains. HuN4 infection resulted in extensive inflammatory cell infiltration, marked alveolar septal thickening, mild hemorrhage, and focal bronchial epithelial degeneration and necrosis, whereas R-HuN4-TNII infection caused comparatively moderate lesions. Similarly, HeB108-infected piglets exhibited inflammatory infiltration and septal widening, while R-HeB108-VGSS infection produced only mild microscopic changes (Fig 5F–J).

Consistent with pulmonary findings, thymic atrophy, a hallmark of PRRSV-2-induced immunosuppression, was more pronounced in piglets infected with HuN4 or HeB108 than in those infected with the corresponding mutant viruses (Fig 5K–O). Overall, these data indicate that the PRRSV-2 mutants exhibit reduced pathogenicity compared with their parental strains.

### Attenuated mutants exhibit reduced replication efficiency *in vivo*

**Fig 6:**
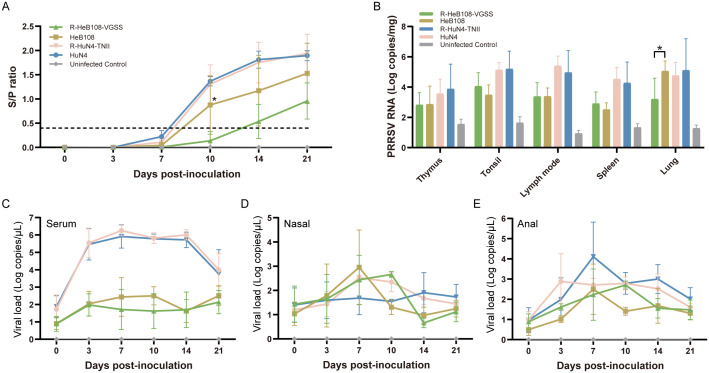
Antibody kinetics and viral distribution in tissues, serum, or swabs. Serum, nasal, and anal swabs were collected at 0, 3, 7, 10, 14, and 21 dpi, whereas tissue samples were collected at 21 dpi. **(A)** PRRSV-2-specific antibodies were measured using the IDEXX ELISA kit. Serum was confirmed to be positive when the S/P ratio>0.4. **(B)** Viral loads in different tissues. **(C)** Viral load in serum. Viral shedding in nasal swabs **(D)** and anal swabs **(E)**. *, *P* < 0.05, significant difference between the R-HeB108-infected and HeB108-infected groups. The data are presented as mean ± *SD*.

To evaluate viral replication and host immune responses in vivo, PRRSV-2-specific antibody kinetics, tissue viral loads, viremia, and viral shedding were assessed. All piglets infected with HuN4 or R-HuN4-TNII seroconverted by 10 dpi (S/P > 0.4), with no significant difference in antibody levels between the two groups (Fig 6A), whereas no antibodies were detected in the control group. In contrast, PRRSV-2-specific antibody responses in HeB108- and R-HeB108-VGSS-infected piglets were delayed, particularly in the R-HeB108-VGSS group. Moreover, antibody titers in R-HeB108-VGSS–infected piglets were significantly lower than those in HeB108-infected piglets, suggesting reduced replication capacity of the mutant virus in vivo. RT-qPCR analysis revealed that viral loads in the lungs of HeB108-infected piglets were significantly higher than those in R-HeB108-VGSS–infected piglets (*P* < 0.05) (Fig 6B), whereas only minor differences were observed between HuN4- and R-HuN4-TNII–infected groups. Viremia was detectable as early as 3 dpi in HuN4- and R-HuN4-TNII–infected piglets, peaking at 7 dpi with an average level of 10⁶ copies/μL (Fig 6C). In contrast, serum viral loads in HeB108- and R-HeB108-VGSS–infected piglets were substantially lower, averaging approximately 10² copies/μL. No significant differences in serum viral loads were observed between mutant- and parental-virus–infected groups. Viral RNA was also detected in nasal and anal swabs, with peak shedding occurring at 7–10 dpi (Fig 6D and 6E). However, no significant differences in shedding levels were observed between mutant and parental virus groups, and overall viral loads in swabs were low (approximately 10³ copies/μL). Uninfected piglets remained negative for PRRSV-2 throughout the experiment.

### R-HeB108-VGSS and R-HuN4-TNII exhibited reduced mutation frequencies in vivo

**Fig 7:**
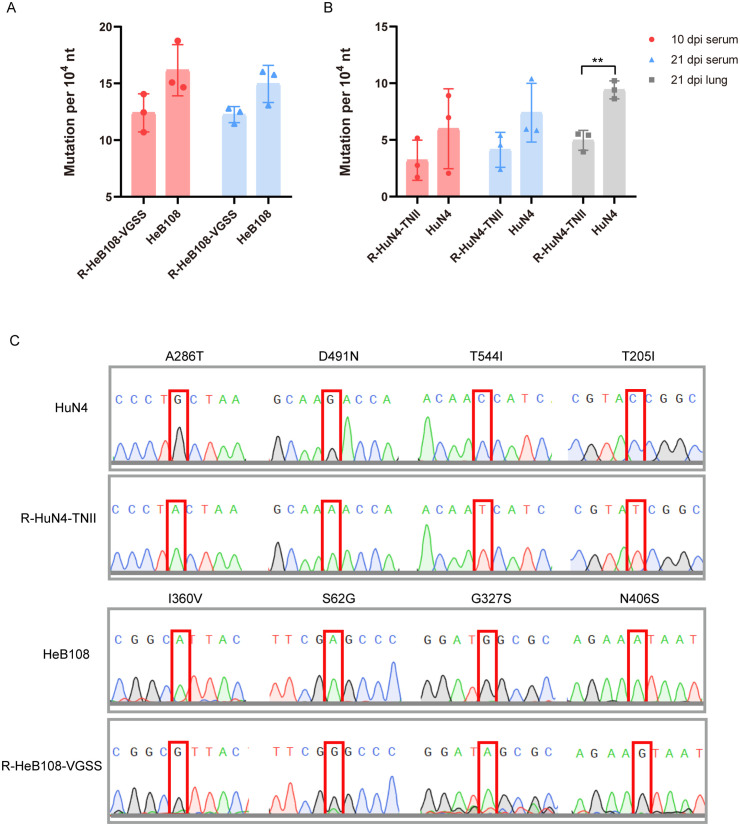
Mutation frequency and genetic stability analysis of R-HeB108-VGSS and R-HuN4-TNII. The mutation frequencies of R-HeB108 **(A)** and R-HuN4 **(B)** and their parental viruses were determined by collecting serum samples at 10 dpi and 21 dpi, and lung tissue samples at 21 dpi, from infected piglets. Approximately 50 monoclonal colonies were sequenced for each sample of ORF3 or ORF5. The mean mutation frequencies (number of nucleotide mutations per 10,000 nucleotides sequenced) ± *SD* represent the averages of all replicates. The same pattern of reduced mutation frequency in the polymerase mutant compared to the parental virus was observed across all replicates. **(C)** The stability of the mutation site was detected at 21 dpi in infected piglets. All mutation sites can exist stably. **, *P* < 0.01, significant difference between the mutant and parental virus.

To investigate the effects of the identified substitutions on viral mutational dynamics, viral mutation frequencies were determined by sequencing the ORF3 and ORF5 genes of R-HeB108-VGSS, R-HuN4-TNII, and parental viruses HeB108 and HuN4 in infected piglets. The nucleotide mutation number from R-HeB108-VGSS and R-HuN4-TNII was lower than that of the parental viruses in serum collected at 10 dpi and 21 dpi (Fig 7A and 7B). Relative to the parental viruses, mutation frequencies were reduced by approximately 1.30-fold and 1.86-fold at 10 dpi and by 1.22-fold and 1.79-fold at 21 dpi for R-HeB108-VGSS and R-HuN4-TNII, respectively. In addition, significantly fewer mutations were detected in the lung tissues of piglets infected with R-HuN4-TNII than in those infected with the parental virus, corresponding to a 1.90-fold reduction in mutation frequency (Fig 7B). To evaluate the genetic stability of the introduced substitutions, viral genomes recovered from infected piglets were sequenced after multiple rounds of in vivo replication. No reversion mutations were detected in either R-HeB108-VGSS or R-HuN4-TNII (Fig 7C), indicating that the engineered substitutions remained genetically stable during infection. These data indicate that R-HeB108-VGSS and R-HuN4-TNII exhibit increased replication fidelity compared to their respective parental viruses.

### Ribavirin-induced mutants showed lower fitness *in vitro*

**Fig 8:**
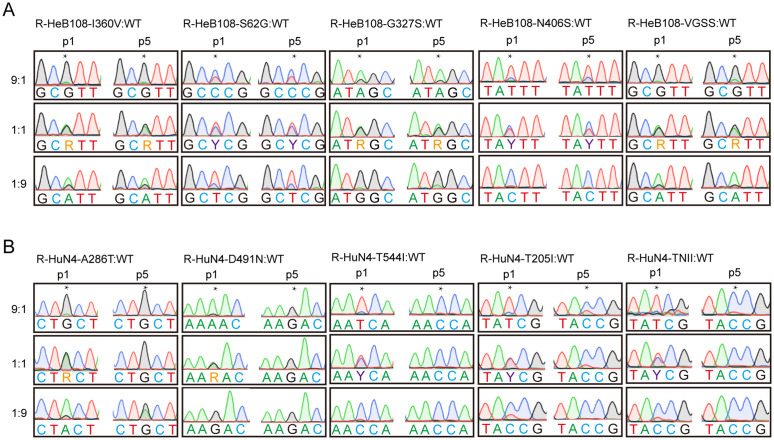
Competition assay of mutations in vitro. **(A)** R-HeB108-I360V, R-HeB108-S62G, R-HeB108-G327S, R-HeB108-N406S, or R-HeB108-VGSS mutants were mixed with the parental virus HeB108 at a ratio of 9:1, 1:1, or 1:9, respectively; **(B)** R-HuN4-A286T, R-HuN4-D491N, R-HuN4-T544I, R-HuN4-T205I, or R-HuN4-TNII mutants were mixed with the parental virus HuN4 at a ratio of 9:1, 1:1, or 1:9, respectively. The mixed mutant virus and the parental virus were inoculated into Marc-145 cells at an MOI of 0.1, and the progeny viruses were passaged in Marc-145 cells for 5 passages. Subsequently, the nsp9 or nsp10 region was sequenced. The abundance of each competitor was measured as the height of the nucleotide peak for the mutant or the parental virus in sequencing chromatograms. *, represents the mutation site.

To evaluate the fitness of the mutant viruses, a direct competition assay was performed by co-culturing the mutant and parental viruses. If a virus becomes dominant in the viral population, it is likely to have higher cellular fitness, and vice versa. Fitness of each mutant and the parental virus was examined in Marc-145 cells. The chromatogram peak height showed no significant differences between the R-HeB108 mutants and the parental virus HeB108 (Fig 8A), indicating that the R-HeB108 mutants have excellent adaptability to Marc-145 cells. Interestingly, the chromatogram peak heights for the R-HuN4 mutants were significantly lower than those of the parental virus HuN4 (Fig 8B), indicating that all R-HuN4 mutants exhibit a fitness disadvantage relative to HuN4.

### The resistance of PRRSV-2 mutants to nucleoside analogues

**Fig 9:**
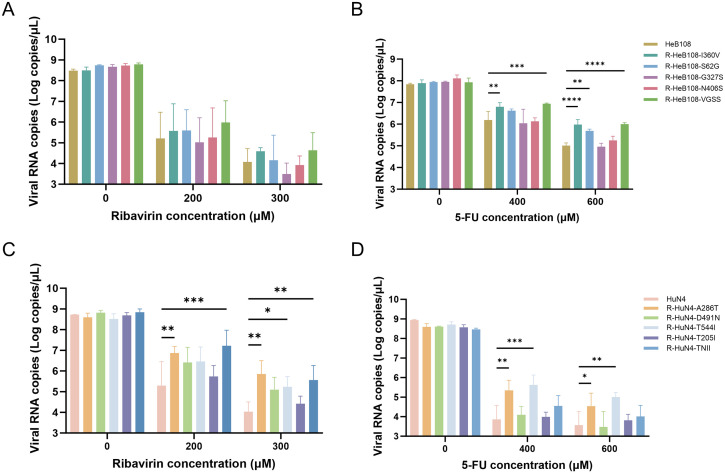
Viral sensitivity to ribavirin or 5-FU. HeB108 and its ribavirin-associated mutants were inoculated into Marc-145 cells that were pre-treated with ribavirin **(A)** or 5-FU **(B)** at an MOI of 0.01 for 72 h. HuN4 and its ribavirin-associated mutants were inoculated into Marc-145 cells that were pre-treated with ribavirin **(C)** or 5-FU **(D)**. Cells without ribavirin or 5-FU treatment served as a control to evaluate the virus titers. Viral genomic RNA level was determined by RT-qPCR. *, *P*  <  0.05; **, *P*  <  0.01; ***, *P*  <  0.001; ****, *P*  <  0.0001.

The ribavirin-selected mutants and their parental viruses were inoculated into Marc-145 cells and treated with ribavirin or 5-FU, with a drug-free control group included for comparison. In the absence of the drug, no significant differences in replication levels were observed among the mutant viruses. However, in the presence of 300 μM ribavirin, R-HeB108-VGSS and R-HeB108-I360V grew well (Fig 9A). Similarly, R-HeB108-VGSS, R-HeB108-S62G, and R-HeB108-I360V showed apparent resistance to 5-FU (Fig 9B). Among HuN4-derived mutants, R-HuN4-TNII, R-HuN4-A286T and R-HuN4-T544I showed resistance to ribavirin (Fig 9C), whereas R-HuN4-A286T and R-HuN4-T544I were resistant to 5-FU (Fig 9D). Thus, these mutations are associated with nucleoside analogue resistance, which could increase the polymerase fidelity.

### Ribavirin-induced PRRSV-2 mutants showed recombination deficiency

**Fig 10:**
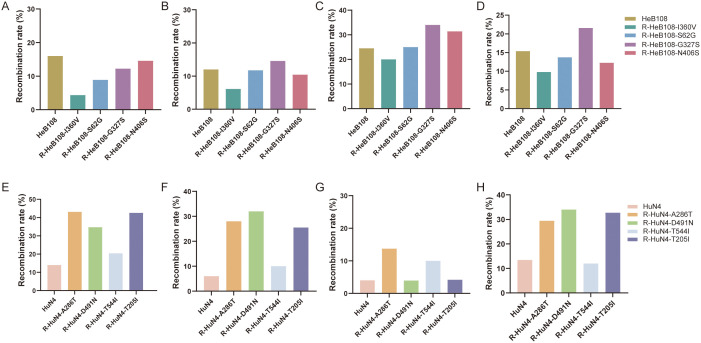
The recombination frequency of mutant viruses in PAMs. **(A–D)** The recombination frequency of R-HeB108 mutant virus was detected in co-infection assay with HuN4. **(E–H)** The recombination frequency of R-HuN4 mutant virus co-infected with HeB108. The experiments were performed in four replicates.

To investigate whether the substitution affected viral recombination, the mutant viruses R-HeB108-I360V, R-HeB108-S62G, R-HeB108-G327S, and R-HeB108-N406S were each separately co-infected with HuN4 in PAMs. Similarly, the mutant viruses R-HuN4-A286T, R-HuN4-D491N, R-HuN4-T544I, and R-HuN4-T205I were each separately co-infected with HeB108. The results showed that the recombination rate of R-HeB108-I360V was lower than that of the parental virus, HeB108, during co-infection with four different repeats. The recombination rate of R-HeB108-N406S was slightly lower than that of the parental virus HeB108 in three of the four replicates (Fig 10A–D). However, the recombination frequency of HuN4-derived mutants was usually higher than that of their parental virus HuN4 (Fig 10E–H).

### The I360V substitution in nsp9 reduces the recombination frequency of PRRSV-2

**Fig 11:**
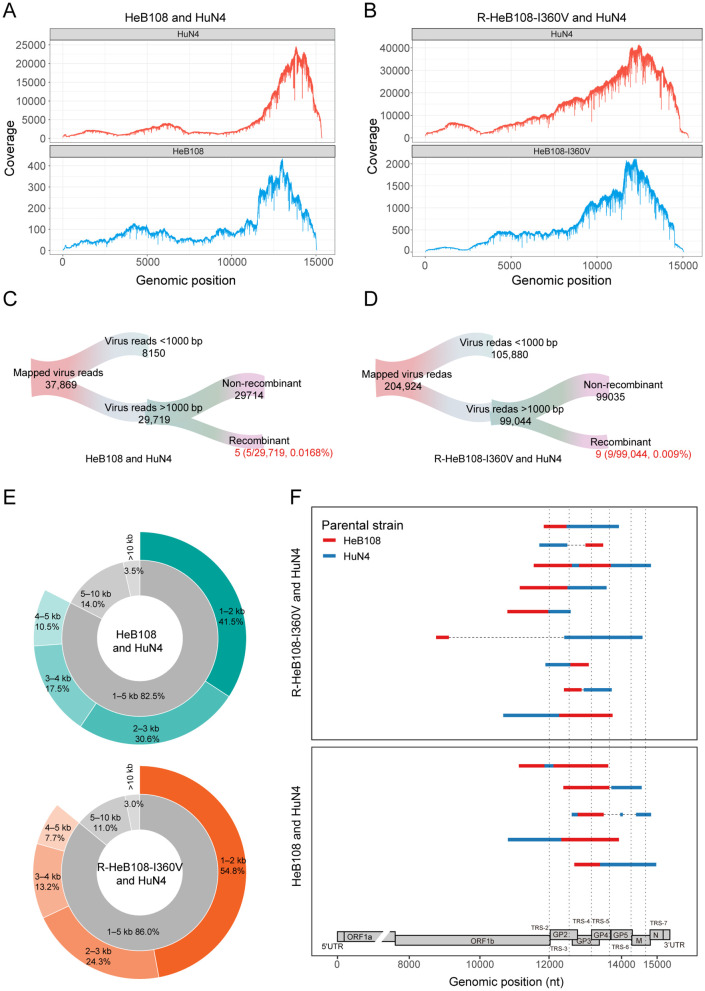
Nanopore sequencing analysis after co-infection of Marc-145 cells with R-HeB108-I360V and R-HuN4. Sequencing depth analysis of the co-infection of the parental virus HeB108 **(A)** and R-HeB108-I360V mutant virus **(B)** with HuN4 strain. Analysis of recombination frequency between HeB108 **(C)** and R-HeB108-I360V **(D)**. **(E)** Length distribution of viral reads longer than 1 kb. **(F)** Schematic diagram illustrating the mapping of recombinant reads to the viral genome. Each recombinant RNA molecule is aligned to the HuN4 reference genome, with gene boundaries annotated in gray below. Sequences are colored according to their parental origin (red, HeB108; blue, HuN4). Horizontal black dotted lines indicate deleted regions, and vertical black dashed lines denote the positions of genomic transcription regulatory sequences (TRSs).

With advances in sequencing technologies, the emergence of nanopore sequencing has enabled the accurate identification of viral recombination events and the generation of long reads spanning recombination junctions. Previous studies have successfully used nanopore sequencing to analyze recombination frequencies among coronaviruses (39). Therefore, to more accurately assess the effect of mutation sites on recombination, we selected the R-HeB108-I360V strain as a representative and measured changes in recombination frequency using nanopore sequencing and bioinformatic analysis. R-HeB108-I360V and HuN4 strains were co-infected into Marc-145 cells, and total RNA was extracted at 24 hpi for long-read sequencing. Sequencing data were analyzed using the NanoSort pipeline [39] to quantify recombination events. Both datasets exhibited markedly higher sequencing depth toward the 3′ end of the genome (Fig 11A and 11B), likely reflecting the nested transcription strategy of PRRSV-2 sub-genomic RNAs [7]. In the co-infected samples, HuN4-derived reads were more abundant than those from HeB108 (Fig 11A and 11B), indicating competitive replication between the two parental viruses. Despite the dominance of HuN4, recombinant RNAs were readily detected. This result revealed that the parental virus HeB108 exhibited a recombination frequency of 0.0168% (Fig 11C), whereas R-HeB108-I360V showed a decreased frequency of 0.009% (Fig 11D), corresponding to a 1.87-fold reduction.

Considering that recombinant reads were identified from reads longer than 1,000 nt, we first examined the length distribution of > 1, 000 nt reads in greater detail. In both datasets, the majority of reads were within the 1–5 kb range; however, R-HeB108-I360V has a higher proportion in the 1–2 kb range compared with the parental virus (Fig 11E). We next mapped recombinant reads onto the viral genome and found that these recombination-associated reads were also predominantly 1–5 kb in length (Fig 11F), consistent with the overall read length distribution shown in Fig 11E. Overall, these results demonstrate that the I360V substitution in nsp9 significantly reduces PRRSV-2 recombination frequency, in agreement with the results obtained from Sanger sequencing analyses.

## Discussion

Recombination is a major driving force of viral evolution, particularly in RNA viruses. Recombination plays a pivotal role in the evolution of PRRSV-2, driving genetic diversity and adaptation in swine populations. Both NADC30-like and NADC34-like PRRSV-2 belong to L1, which are characterized by a high recombination rate and a distinct amino acid deletion pattern in nsp2. Whereas HP-PRRSV, which belongs to L8, is characterized by high fever, morbidity, and mortality [21]. The NADC30-like PRRSV-2 and HP-PRRSV recombination produced novel recombinant strains with altered pathogenicity in pigs [14,15,40]. Notably, most of these recombinants use HP-PRRSV and NADC30-like PRRSV-2 as parental viruses, with some also involving PRRSV-2 MLV vaccines.

Here, the PRRSV-2 L1 and L8 representative strains HeB108 and HuN4 were passaged under ribavirin pressure. Ribavirin is a nucleoside analogue that can efficiently suppress replication of porcine nidoviruses, including PRRSV and PEDV [41]. Given the key role of nsp9 and nsp10 in viral replication, we conducted an in-depth analysis of amino acid substitutions in these two nonstructural proteins. Results showed that amino acid substitutions in nsp9 and nsp10 increased viral polymerase fidelity and reduced the possibility of recombination. The rescued ribavirin-associated mutants, R-HuN4-A286T, R-HuN4-T544I, R-HuN4-TNII, R-HeB108-I360V, R-HeB108-S62G, and R-HeB108-VGSS, showed resistance to high concentrations of ribavirin or 5-FU. Polymerase fidelity and pathogenicity to piglets of R-HeB108-VGSS, R-HuN4-TNII, and their parental viruses, HeB108 and HuN4, were evaluated *in vivo*. Compared with HeB108 and HuN4, piglets infected with R-HeB108-VGSS and R-HuN4-TNII showed attenuation phenotype, and amino acid substitutions in nsp9 and nsp10 were stable in infected piglets. Notably, the mutant viruses R-HeB108-VGSS and R-HuN4-TNII exhibited reduced mutation frequencies *in vivo*. The extent of fidelity alteration likely varies among different amino acid substitutions. Comparing the biochemical kinetics of viral polymerases during RNA synthesis represents the most direct approach to assess these differences. Unfortunately, we were unable to obtain in vitro‑expressed nsp9 protein with detectable polymerase activity. Moreover, the current incomplete understanding of the RTC assembly mechanism of PRRSV-2 has hampered the establishment of a stable in vitro extension assay, which remains a challenge in the field.

A previous study has identified key amino acids in the RdRp of PRRSV-2 that can affect fidelity and pathogenicity. Specifically, Tian et al. identified two nsp9 substitutions under ribavirin pressure, A283T and H421Y, which play a crucial role in viral replication by affecting the polymerase fidelity [42]. In the present study, we identified the corresponding substitution (A286T; equivalent to A283T after alignment). This numbering discrepancy likely results from differences in nsp9 N-terminal annotation, leading to a + 3 offset for the same physical residue after sequence alignment. The presence of this residue in different strains suggests a conserved role in ribavirin resistance. In contrast, the H421Y substitution reported in Ref 43 was not detected in our study, most likely due to differences in viral genetic background. Ref 43 used PRRSV-2 strains VR2385 and MN184B, whereas our study employed the HuN4 and HeB108 strains. Different PRRSV-2 lineages may exhibit distinct adaptive responses under ribavirin selection pressure due to differences in their genetic backgrounds, thereby leading to different amino acid substitutions associated with viral adaptation or resistance. Similarly, amino acids at positions 519 and 544 in nsp9 are involved in HP-PRRSV replication efficiency, thereby contributing to its enhanced pathogenicity [38]. nsp10 is also involved in recombination events in virulent strains [43,44], and mutations in the nsp10 gene may lead to the formation of novel virulent strains [45]. Collectively, the HP-PRRSV nsp9- and nsp10-coding regions are closely associated with replication efficiency *in vitro* and *in vivo*, as well as with increased pathogenicity and fatal virulence in piglets [46]. Therefore, this study focused on amino acid substitutions in nsp9 and nsp10.

Previous studies have also shown that viral polymerases and helicases affected viral recombination [31–33,35,36]. In PRRSV-2, nsp9 encodes RdRp, which affects viral replication [47–49], whereas nsp10 encodes a helicase with ATPase activity and unwinds double-stranded RNA [50]. The viral RdRp could use a template-switching mechanism to catalyze RNA recombination. Recent studies have shown that complete inactivation of internal ribosome entry site-mediated translation of the donor enteroviral genome enhances recombination [51]. Some external factors can also affect viral recombination, such as host cellular RNA helicases, which can influence RNA recombination and viral replication [52,53]. Thus, viral RNA recombination is a complex interplay between intrinsic RdRp template-switching activity, translational regulation of viral genomes, and host-derived RNA helicases.

Because ribavirin-resistant PRRSV-2 mutants exhibit a high-fidelity phenotype, it is hypothesized that increased polymerase fidelity could reduce viral recombination rates. In this study, ribavirin-selected mutants and their parental viruses were co-infected in PAMs, and the results showed that R-HeB108-I360V and R-HuN4-T544I had lower recombination rates than their parental viruses. We also observed that the proportion of parental viruses produced during co-infection varied significantly. Therefore, the low recombination rate of the mutant virus could be due to poor competitiveness during co-infection. Due to the impaired replication capacity of the mutant virus, co-infection with its parental strain resulted in significantly lower progeny production of the mutant. This reduced fitness largely restricted the potential of the mutant to engage in recombination events compared with the parental virus [54–56]. The underlying mechanisms responsible for this low recombination frequency therefore warrant further investigation.

The results of multiple recombination experiments suggested that recombination is highly random. To more accurately assess recombination rates, we attempted to establish a PRRSV-2 cell culture-based recombination assay using multiple combinations of replication-defective and structural gene-deficient constructs, an approach that has been widely applied in recombination study of picornaviruses [31]. However, despite repeated optimization attempts, no recombinant virus could be successfully recovered. We speculate that the rapid replication kinetics of picornaviruses, combined with the high transfection efficiency of BHK cells used for virus rescue, facilitates the faster rescue of virus from the infectious clone. However, quantitative analysis of recombination frequency in PRRSV-2 remains challenging. Unlike picornaviruses, which can be readily recovered and characterized following reverse genetic manipulation, PRRSV-2 exhibits slower replication kinetics and typically requires additional amplification after rescue to generate sufficient viral populations. These experimental constraints make it challenging to accurately quantify recombination frequencies directly from newly rescued viruses.

Advances in nanopore sequencing have enabled quantitative analyses of viral recombination frequencies. By combining long-read sequencing with the NanoSort algorithm, a previous study estimated an average recombination frequency of 0.025% between canine coronavirus and feline coronavirus [39]. Using a similar analytical framework, we calculated the recombination frequency between the R-HeB108-I360V strain and the HuN4 strain to be 0.009%, whereas the recombination frequency between the parental viruses, the HeB108 strain, was 0.0168%. These results indicate that the I360V substitution in nsp9 enhances polymerase fidelity and significantly reduces the occurrence of recombination events. Previous studies in coronaviruses have shown that subgenomic RNAs can serve as templates for additional rounds of replication and recombination [57], raising the possibility that similar processes may occur in other nidoviruses. Because the nanopore reads generated in this study did not span complete PRRSV-2 genomes, we could not definitively distinguish whether the detected recombination events originated from genomic or subgenomic RNAs. Importantly, this limitation does not affect the observed recombination frequencies reported in our study. Our findings nevertheless suggest that PRRSV-2 replication is accompanied by extensive RNA recombination activity that may contribute to viral genetic diversity. Whether these recombination products can transmit within swine populations remains unclear. Future studies combining full-length long-read sequencing, reverse genetics, and *in vivo* transmission models will be required to distinguish transient replication intermediates from heritable recombinant genomes and determine their contribution to PRRSV-2 evolution.

Previous studies have shown that increasing RdRp fidelity attenuates poliovirus by reducing its ability to enter the brain and cause disease [58,59]. Polymerase fidelity also contributes to pathogenicity and transmissibility in vivo for foot-and-mouth disease virus [60,61]. In addition, ribavirin-resistant PRRSV-2 mutants (RVRp22) are attenuated in pigs and retain this phenotype [62]. This study obtained a similar conclusion; the ribavirin-selected mutants R-HeB108-VGSS and R-HuN4-TNII were attenuated in pigs compared to their parental viruses. Furthermore, we identified key amino acids in RdRp and helicases that affected PRRSV-2 pathogenicity, indicating that PRRSV-2 polymerase fidelity influences its pathogenicity. The mutant viruses R-HeB108-VGSS and R-HuN4-TNII were stable in piglets without reversion. These data demonstrated that modulated replication fidelity attenuated viruses in animals, a desirable property for developing genetically stable vaccine candidates.

PRRSV-2 exhibits complex genetic diversity, in part because it is prone to recombination. Vaccination has been the primary method for preventing and controlling PRRS. However, owing to the rapid mutation and extensive recombination of PRRSV-2, PRRS MLV carries the potential risk of virulence reversion due to recombination. Recently, recombination between the MLV and wild-type PRRSV-1 or PRRSV-2 strains has been reported [15,16]. The ribavirin-resistant method can be used to develop a safer PRRS MLV. PRRSV-2, especially NADC30-like PRRSV-2, are prone to recombination, possibly because of the influence of certain amino acids in nsp9 or nsp10, which encode RdRp and helicase, respectively, which may affect the process of viral replication. This is attributed to the crucial roles of nsp9 and nsp10 in PRRSV-2 replication. Most RNA viruses recombine via a copy-choice mechanism, during which RdRp switches from a donor to an acceptor template, resulting in the formation of recombinant RNA molecules [63]. This event usually occurs during virus replication. nsp9 encodes an RNA replicase of PRRSV-2, which is closely related to PRRSV replication [38]. PRRSV-2 helicase nsp10 is an important component of the viral replication-transcription complex [64]. It has also been confirmed in other studies that nsp9 and nsp10 are crucial enzymes for viral RNA synthesis [9,37,65].

In conclusion, this study identified key amino acids in RdRps and helicases that affect PRRSV-2 polymerase fidelity, pathogenicity, and recombination rate. These specific amino acids could lay the foundation for the development of PRRS MLVs, which are stable, safe, and less prone to recombination. These findings may contribute to the development of a new type of vaccine through rational design.

## Materials and methods

### Ethic statement

The pathogenicity examination was approved by the Ethics Committee of Harbin Veterinary Research Institute, Chinese Academy of Agricultural Sciences (Approval Number: 220902–03).

### Cells and viruses

Marc-145 cells were cultured in DMEM (Sigma, Germany) supplemented with 10% fetal bovine serum (FBS) and 1% penicillin-streptomycin. Primary porcine alveolar macrophages (PAMs) were isolated from the lung lavage of four-week-old specific pathogen-free piglets. PAMs were cultured in RPMI 1640 medium (Thermo Fisher Scientific, USA) supplemented with 10% FBS and 1% penicillin-streptomycin. L1 NADC30-like PRRSV-2 HeB108 strain (GenBank accession no. MN046224), L8 PRRSV-2 HuN4 strain (GenBank accession no. EF635006), and their infectious clones were preserved in our laboratory.

### Determining the range of ribavirin concentrations

The cytotoxic effects of ribavirin on Marc-145 cells were analyzed as previously described [58]. Briefly, Marc-145 cells were treated with different concentrations of ribavirin (0, 100, 200, 300, 400, 500, and 600 μM) for 72 h, and the optimal nontoxic ribavirin concentration that moderately reduced virus titers was determined through the cell viability (S1 Fig). Marc-145 cells were pre-treated with ribavirin for 2 h at the same concentration range as described above. Subsequently, the cells were infected with HuN4 at a multiplicity of infection (MOI) of 0.1 for 2 h. The inoculated virus was aspirated, and medium containing optimal ribavirin concentrations was added. The cells were incubated until they exhibited a cytopathic effect (CPE). The viral titer and RNA copy number were determined to evaluate the antiviral effects. Ultimately, ribavirin concentrations that reduced virus titers by 0.5-2 logs (compared to the untreated control) but were not highly toxic to the cells were determined.

### Serial passaging of HuN4 and HeB108 in the presence of ribavirin

Ribavirin-resistant mutants were generated by serial passaging of PRRSV-2 HeB108 and HuN4 in Marc-145 cells with gradually increasing concentrations of ribavirin. The concentration of ribavirin (250 μM) that reduced virus titers was determined by tissue culture infectious dose (TCID_50_) and quantitative reverse transcription PCR (RT-qPCR) (S1 Fig). Briefly, Marc-145 cells seeded in a 12-well plate were treated with 250 μM ribavirin for 2 h, followed by medium removal; the cells were inoculated with HeB108 or HuN4 at an MOI of 0.1, respectively. The supernatant was discarded after 1 h of inoculation, and the cells were washed thrice with PBS; subsequently, fresh medium supplemented with 250 μM ribavirin was added. The viruses were harvested at 72 hours post-inoculation (hpi) and used for inoculation in subsequent passages. The concentration of ribavirin was maintained at 250 μM for passages 1–10, 300 μM for passages 11–20, and 350 μM for passages 21–30. PRRSV-2 HuN4 and HeB108 were simultaneously passaged in Marc-145 cells without ribavirin as untreated controls. Viral RNA was extracted from the P10, P15, P20, P25, and P30 viruses. Viral nsp9 (encoding RdRp) and nsp10 (encoding helicase) were amplified using reverse transcription PCR (RT-PCR) with primers (S3 Table). PCR products were purified and inserted into a pMD-18T vector (TaKaRa, China) for DNA sequencing. The Lasergene software package (DNASTAR Inc.) was used for sequence alignment and analysis.

### Construction and rescue of mutant viruses

To investigate the potential contributions of mutated amino acids during serial passaging with ribavirin, specific amino acid residues at positions 286, 491, 544, and 360 of nsp9 and positions 205, 62, 327, and 406 of nsp10 were swapped individually in infectious clones of PRRSV-2 HeB108 or HuN4 by site-directed mutagenesis with the corresponding primers (S3 Table). Site-directed mutagenesis, construction, and rescue of the mutant viruses were performed as previously described [66]. Specifically, the mutations I360V in nsp9 and S62G, G327S, and N406S in nsp10 were individually introduced into infectious clone HeB108 (S1 Table), and the mutations A286T, D491N, T544I in nsp9 and T205I in nsp10 were individually introduced into the infectious clone HuN4 (S2 Table). In addition, combined mutations in HeB108-VGSS and HuN4-TNII were generated. These site-directed mutant infectious clone plasmids were validated by DNA sequencing. Marc-145 cells at 80% confluence were seeded in six-well plates, and 4 μg of each mutant plasmid was transfected into Marc-145 using X-treme Gene HP DNA reagent (Roche, Germany) according to the manufacturer’s instructions. At 96 hpi, the rescued viruses were identified using an indirect immunofluorescence assay (IFA) with a monoclonal antibody (3F7) against the M protein of PRRSV-2 [67]. All the generated viruses were further propagated in Marc-145 cells for 10 passages, and the stability of the introduced mutations was confirmed by sequencing.

### Viral replication kinetics in Marc-145 cells

To compare the replication kinetics of mutant viruses and their respective parental viruses, viral titers were measured using a microtitration assay in 96-well plates and calculated as 50% TCID_50_/mL. Subsequently, Marc-145 cells grown in 12-well plates were infected with viruses at an MOI of 0.01, incubated for 1 h at 37 °C, the supernatants were discarded, and cells were washed thrice with PBS, overlaid with DMEM supplemented with 2% FBS, and incubated at 37 °C. Cells were harvested at 0, 24, 48, 72, 96, and 120 hpi. Virus titers were measured and calculated as TCID_50_. In addition, total RNA was extracted, and RT-qPCR was used to quantify viral copy numbers, as previously described [68]. To assess the replication kinetics of the mutant viruses in the presence of 300 μM ribavirin, Marc-145 cell monolayers were pre-treated with 300 μM ribavirin for 2 h, followed by infection with either mutant or parental viruses at an MOI of 1. After a 2‑hour adsorption period, the cells were washed with PBS and then cultured in medium containing 2% FBS and 300 μM ribavirin at 37°C in a 5% CO_2_ incubator. Samples were collected at the indicated time points to determine viral titers and copy numbers. Mean values and standard deviations were calculated from three independent experiments.

### Piglet infection experiment

Twenty-five four-week-old SPF piglets were purchased from the National Science and Technology Infrastructure Center (Harbin, China). Piglets were randomly divided into five groups (n = 5 per group) and raised in separate biological safety level II rooms. Piglets in groups 1#-4# were inoculated with R-HeB108-VGSS, HeB108, R-HuN4-TNII, or HuN4. Each piglet was inoculated intranasally (1 mL per nostril) or intramuscularly (1 mL). The viral titers of R-HeB108-VGSS and HeB108 were 10^5^ TCID_50_/mL, whereas those of R-HuN4-TNII and HuN4 were 10^4^ TCID_50_/mL. Piglets in group 5# were inoculated with the same volume of DMEM via the same route. Each piglet’s clinical symptoms and rectal temperature were monitored and scored daily until 21 days post-infection (dpi) using a scoring system from a previous study [46]. The body weights of the piglets were measured at 0 and 21 dpi. Blood, nasal swabs, and anal swabs were collected from the piglets at 0, 3, 7, 10, 14, and 21 dpi. All piglets were euthanized at 21 dpi and necropsy was performed to examine the immunological effects and pathological lesions further. Lungs, inguinal lymph nodes, spleen, tonsils, and thymus were collected for viral load examination, and lung tissues from piglets were fixed in 4% paraformaldehyde for Hematoxylin and eosin staining.

### Viral load and antibody level of infected piglets

RT-qPCR was used to determine the viral load in different tissues, serum, and swabs from infected piglets, as previously described [69]. PRRSV-2 antibodies in swine serum were determined using a HerdCheck PRRS 2XR ELISA Kit (IDEXX, USA) according to the manufacturer’s instructions.

### Calculation of viral mutation frequency

To determine viral mutation frequencies, viral RNA was extracted from serum at 10 and 21 dpi and from lung tissue samples at 21 dpi. The high-fidelity PCR enzyme KOD-plus (TOYOBO, Japan) was used to amplify the full-length ORF3-ORF5 sequence using the primers listed in S3 Table. Gel-purified PCR products were cloned into the pMD18-T vector for sequencing. Owing to the high mutation frequency of ORF3 and ORF5, approximately 40–50 clones of ORF3 or ORF5 gene sequences of approximately 600 nt per replicate were sequenced for each population. Sequences were assembled and analyzed using the Lasergene software package. Mutation frequencies (mutations per 10,000 nt) were determined as described previously [58]. The number of mutations per 10^4^ nucleotides sequenced was determined as the total number of mutations identified in each population divided by the total number of nucleotides sequenced for that population multiplied by 10^4^.

### Location of ribavirin-resistance-related mutations

To analyze the positions of ribavirin-associated amino acid substitutions within the spatial structures of PRRSV-2 nsp9 and nsp10, their 3D structures were predicted using the Swiss Model server (www.swissmodel.expasy.org/). The amino acid sequences of nsp9 and nsp10 were searched for homology in the Protein Data Bank. The Swiss model was used to construct a 3D model, and the best models were selected based on the analysis results of the internal scoring functions of the protein modeling module and the Profile-3D program. Arrows indicate mutation sites. In addition, 15 representative PRRSV-2 sequences collected from GenBank were selected to compare amino acid substitutions screened by ribavirin pressure. Fifteen representative PRRSV-2 reference strains were collected from GenBank, selected based on two criteria: the first-isolated strain of each lineage, or strains with clearly characterized biological properties.

### Direct competition assays

Site-directed mutated or parental viruses were mixed in three different ratios (9:1, 1:1, and 1:9) and used to infect Marc-145 cells at an MOI of 0.1. At 48 hpi, 100 µL of culture supernatant from infected cells was extracted and used to inoculate new Marc-145 cells for subsequent passages. Five passages were performed, and the viral RNA from passages 1 (P1) and 5 (P5) was extracted from the inoculum using the RNeasy Mini kit (Qiagen, Germany). The nsp9 and nsp10 genes were amplified by RT-PCR, and the PCR amplicons were sent for Sanger sequencing. The abundance of each competitor was measured as the peak height of the nucleotide corresponding to the parental virus or mutant sequence in the sequencing chromatogram, as previously described [31].

### Sensitivity to nucleoside analogs

Monolayers of Marc-145 cells were pre-treated with 100, 200, 300, and 350 μM ribavirin, or 400 and 600 μM 5-fluorouracil (5-FU) (MCE, China) for 2 h. The cells were infected with site-directed mutated or parental virus at an MOI of 0.01 for 2 h. The infected cells were washed three times with PBS. Subsequently, the cells were cultured with DMEM medium supplemented with 2% FBS and 250 μM ribavirin or 400 μM 5-FU and incubated at 37 °C in 5% CO_2_ for 72 h. The viruses were harvested and analyzed by RT-qPCR as described above. The experiments were independently repeated three times.

### Recombination rate detection

A viral recombination assay was performed by co-infecting PAMs with the mutant and parental viruses. The mutant viruses R-HuN4-A286T, R-HuN4-D491N, R-HuN4-T544I, and R-HuN4-T205I were co-infected with HeB108 in PAMs at a ratio of 3.5:1. Similarly, mutant viruses R-HeB108-I360V, R-HeB108-S62G, R-HeB108-G327S, and R-HeB108-N406S were co-infected with HuN4 in PAMs at a ratio of 1:5. After 1 h of infection, the viral supernatant was discarded and cells were washed with PBS. Subsequently, the cells were supplemented with RPMI 1640 medium containing 2% FBS and cultured at 37 °C for 24 h. Subsequently, the viral supernatant was absorbed, viral RNA was extracted, and RT-PCR was performed to amplify the ORF2, ORF3, and ORF4 regions, as these regions have higher mutation frequencies [70]. Amplified PCR products were purified and cloned into the pMD18-T vector. For each population, approximately 40–50 colonies were selected for sequencing. Four independent replicates were analyzed for each mutant. Simplot and RDP4 were used to analyze recombination in the obtained sequences, using methods described in a previous study [71]. The recombination frequency was calculated by comparing the number of recombinant clones with the total number of sequenced clones.

### Library construction and sequencing

To assess the impact of the selected mutation on viral recombination, the R-HeB108-I360V and HuN4 strains were co-infected, and long-read sequencing was used to quantify recombination events directly. Briefly, Marc-145 cells were co-infected with PRRSV-2 strains HeB108 and HuN4, or R-HeB108-I360V and HuN4, at a total MOI of 1 (0.5 per virus). Cells and supernatant were collected into Trizol for RNA extraction at 24 hpi. Samples were then sequenced using the nanopore sequencer, the CycloneSEQ platform (CycloneSEQ Tech, China), which was conducted at Chengdu Phagetimes Biotech Co., Ltd.

### Alignment and analysis

Long-read sequencing data were first quality filtered using NanoFilt (v 2.8.0) with the parameters “-q 7-l 1000” to retain reads longer than 1,000 nt with an average quality score greater than Q7. The filtered reads were then analyzed using the NanoSort algorithm [39], which is designed to identify recombinant reads by assessing parental origin along individual long-read sequences. Briefly, the NanoSort algorithm uses seqkit (v0.11.0) to split each long read into 200 nt subreads with a 10 nt sliding window. Each sub-read is independently aligned to the parental reference genomes using minimap2 (v2.23). Based on the alignment results, subreads are classified as originating from parental strain 1 or parental strain 2 using a likelihood-based scoring framework.

Because NanoSort requires parental likelihood models as input, the filtered reads were first aligned separately to the HeB108 and HuN4 reference genomes using minimap2. Reads that uniquely mapped to a single parental genome were extracted and used to construct strain-specific trinucleotide frequency tables. Parental likelihood scores were then calculated using a custom Python script. Finally, recombinant reads were identified by running the NanoSort command: *nanosort.sh HuN4 HeB108 test_reads.fastq.gz*.

### Statistical analysis

All data are expressed as means ± standard deviation (*SD*). GraphPad Prism v8.31 (GraphPad Software Inc.) was used for statistical analysis, and statistical significance was determined using one-way and two-way analysis of variance. A value of *P* < 0.05 was considered statistically significant.

## Supporting information

S1 Fig Determining the optimal conditions to select for PRRSV-2 mutagen resistance. Marc-145 were treated with indicated concentrations of ribavirin (A) or 5-FU (B), and infected with PRRSV-2 HuN4 at a MOI of 0.01. The percentage of cells surviving treatment at 48 hours, determined by Trypan blue staining. 48 hours post-infection, progeny virus was harvested, and titers were determined by RT-qPCR and TCID_50_. (A) and (B) showed Marc-145 cell viability after treatment with ribavirin or 5-FU. (C and D) The ribavirin concentrations of 250 μM reduce virus titers without affecting cell viability. (E) The 5-FU concentrations of 400 μM reduce virus titers without affecting cell viability.
(TIF)

S1 Table The amino acid substitutions in the HeB108 strain of PRRSV-2 selected with ribavirin. (DOCX)

S2 Table The amino acid substitutions in the HuN4 strain of PRRSV-2 selected with ribavirin. (DOCX)

S3 Table Primer sequences used in this study. (DOCX)
