# Supplementary material for: Single amino acid substitution in RdRp reduces viral recombination frequency of NADC30-like porcine reproductive and respiratory syndrome virus type 2
Source: PLoS Pathog. 2026 Aug 5;22(8):e1014500. doi: 10.1371/journal.ppat.1014500 (PMC13466052; doi:10.1371/journal.ppat.1014500)
Supplement: Supplementary file 1 [file ppat.1014500.s002.docx]

S1 Table. The amino acid substitutions in the HeB108 strain of PRRSV-2 selected with ribavirin.

|  | R-HeB108-P10 | | R-HeB108-P15 | | R-HeB108-P20 | | R-HeB108-P25 | | R-HeB108-P30 | |
| --- | --- | --- | --- | --- | --- | --- | --- | --- | --- | --- |
|  | Nucleotide | Amino acid | Nucleotide | Amino acid | Nucleotide | Amino acid | Nucleotide | Amino acid | Nucleotide | Amino acid |
| nsp9 |  |  |  |  | **A→G (1078)** | **I→V (360)** | A→G (1069) | I→V (357) | A→G (1069) | I→V (357) |
| nsp10 | **A→G (184)** | **S→G (62)** | A→G (184) | S→G (62) | A→G (184) | S→G (62) | A→G (184) | S→G (62) | A→G (184) | S→G (62) |
|  | **A→G (1217)** | **N→S (406)** | A→G (1217) | N→S (406) | A→G (1217) | N→S (406) | - | - | - | - |
|  |  |  |  |  |  |  | **G→-A (979)** | **G→S (327)** | G→-A (979) | G→S (327) |

-, indicates the reverse-mutation occurred at this site
