# Supplementary material for: Single amino acid substitution in RdRp reduces viral recombination frequency of NADC30-like porcine reproductive and respiratory syndrome virus type 2
Source: PLoS Pathog. 2026 Aug 5;22(8):e1014500. doi: 10.1371/journal.ppat.1014500 (PMC13466052; doi:10.1371/journal.ppat.1014500)
Supplement: Supplementary file 2 [file ppat.1014500.s003.docx]

S2 Table. The amino acid substitutions in the HuN4 strain of PRRSV-2 selected with ribavirin.

|  | R-HuN4-P10 | | R-HuN4-P15 | | R-HuN4-P20 | | R-HuN4-P25 | | R-HuN4-P30 | |
| --- | --- | --- | --- | --- | --- | --- | --- | --- | --- | --- |
|  | Nucleotide | Amino acid | Nucleotide | Amino acid | Nucleotide | Amino acid | Nucleotide | Amino acid | Amino acid | Nucleotide |
| nsp9 | **G→A (856)** | **A→T (286)** | G→A (856) | A→T (286) | G→A (856) | A→T (286) | G→A (856) | A→T (286) | G→A (856) | A→T (286) |
|  |  |  |  |  |  |  | **C→T (1631)** | **T→I (544)** | C→T (1631) | T→I (544) |
|  |  |  |  |  |  |  |  |  | **G→A (1471)** | **D→N (491)** |
| nsp10 | **C→T (614)** | **T→I (205)** | C→T (614) | T→I (205) | C→T (614) | T→I (205) | C→T (614) | T→I (205) | C→T (614) | T→I (205) |
