# Supplementary material for: Single amino acid substitution in RdRp reduces viral recombination frequency of NADC30-like porcine reproductive and respiratory syndrome virus type 2
Source: PLoS Pathog. 2026 Aug 5;22(8):e1014500. doi: 10.1371/journal.ppat.1014500 (PMC13466052; doi:10.1371/journal.ppat.1014500)
Supplement: Supplementary file 3 [file ppat.1014500.s004.docx]

S3 Table. Primers used in this study.

| Primers | Sequences (5′–3′) |
| --- | --- |
| HeB108-360F | GCACTGAGCGGCGTTACTCAGGGTT |
| HeB108-360R | AACCCTGAGTAACGCCGCTCAGTGC |
| HeB108-62F | GGGAAAGGTTCGGGCCCACTTGACG |
| HeB108-62R | CGTCAAGTGGGCCCGAACCTTTCCC |
| HeB108-327F | GACCGAGAGGATAGCGCCATCACCAT |
| HeB108-327R | TGGTGATGGCGCTATCCTCTCGGTC |
| HeB108-406F | TGCTAGACAGAAGTAATAAAGAGTG |
| HeB108-406R | CACTCTTTATTACTTCTGTCTAGCAC |
| HuN4-286-F | TCCACTTACCCTACTAAGAATTCTAT |
| HuN4-286-R | CATAGAATTCTTAGTAGGGTAAGTGG |
| HuN4-491-F | CTGTTTCTGCAAAACCAGCTAAAGT |
| HuN4-491-R | ACTTTAGCTGGTTTTGCAGAAACAG |
| HuN4-544-F | CAAAGAAGACAATCATCACAGACTC |
| HuN4-544-R | GAGTCTGTGATGATTGTCTTCTTTG |
| HuN4-205-F | CCCCCTCCCGTATCGGCCCATGGGT |
| HuN4-205R | ACCCATGGGCCGATACGGGAGGGGG |
| ORF234-F | CATTGAACCAACTTTAGGCC |
| ORF234-R | TTTCACTCATCTCGGAAGC |
